# Dr. Cardinal T. Wolsey

**Published:** 1916-11

**Authors:** 


**Dr. Cardinal T. Wolsey,** Howard University of Washington, D. C. 1885, died at the home of Dr. E. F. Hurburt of Buffalo, Sept. 26. He was born in Batavia 67 years ago. He resided at 67 Niagara Street, Buffalo, for many years. He was examiner for the Buffalo Marine Corps recruiting station for 20 years and was a member of a number of fraternal organizations.

